# Home-Based Titration with Duodenal Infusion of Levodopa-Carbidopa Intestinal Gel in People with Parkinson's Disease: An Observational Feasibility Study

**DOI:** 10.1155/2024/5522824

**Published:** 2024-04-08

**Authors:** Trine Hørmann Thomsen, Nick Schou Nielsen, Asher Lou Isenberg, Michael Hougaard Møller, Jesper Bøje Clausen, Inge Mona Schack Frederiksen, Louise Olsen, Mahsa Javidi, Jeanet Vilhelmsen, Marc Klee Olsen, Bo Biering-Sørensen

**Affiliations:** ^1^Movement Disorder Clinic, Department of Neurology, Rigshospitalet, Copenhagen, Denmark; ^2^Department of Brain and Spinal Cord Injuries, Rigshospitalet, Copenhagen, Denmark; ^3^Department of Neurology, Nordsjællands Hospital, Hillerød, Denmark; ^4^The Pain Clinic/CRPS Clinic, Department of Neurology, Rigshospitalet, Copenhagen, Denmark; ^5^The Spasticity Clinic, Department of Neurology, Rigshospitalet, Copenhagen, Denmark

## Abstract

**Background:**

Testing and titration of the right levodopa equivalent dose are usually performed during a hospital admission. However, optimal dose titration in people with Parkinson's disease (PwPs) may depend on home environment, emotional stress, and physical activity of everyday life.

**Objective:**

Firstly, to evaluate the feasibility and safety of a home-based LCIG titration program and patients'/caregivers' satisfaction. Secondly, to identify barriers and facilitators for home-based titration.

**Method:**

This study assesses the feasibility and safety of home-based titration of levodopa duodenal infusions with the use of self-reported evaluation questionnaires with open-ended questions included, registration of total time used, and number of contacts/visits. A telemedicine solution was used to remotely monitor the patients, adjust treatment, and provide support and guidance to patients and caregivers.

**Results:**

Ten of 12 PwPs (5 females and 7 males) completed the total titration program. Eight of the 12 PwPs were dependent on help. These 8 PwPs also had a high burden of nonmotor symptoms (NMS). Cognitive impairments varied in severity (range 16–30). Time spent with home visits was on average 93.4 minutes (ranging from 35 to 180 minutes), and the length of the total titration (LCIG initiation to termination of titration) was on average 3.4 days with 2–5 (mean 3.2) contacts/visits with PD team members. The average score on the satisfaction evaluation questionnaires was lower in the caregiver group (mean 31.8) than the PwP outcome (mean 36.2).

**Conclusions:**

Telehealth-assisted home-based titration programs are feasible due to the length of the titration period, number of contacts, and time spent in PwPs' private homes, are rated satisfactory and safe by PwPs and caregivers, and may be a substitute for in-hospital treatment. Clinical recommendations including facilitators and barriers from a patient/caregiver perspective are displayed. This trial is registered with NCT4196647.

## 1. Introduction

Previous studies of duodenal infusion of levodopa on people with Parkinson's disease (PwPs) have reported beneficial effects on motor symptoms and nonmotor symptoms (NMS) [1]. In Denmark, the testing and titration of the right levodopa equivalent dose are usually performed during a hospital admission over a week. However, real-life optimal dose adjustment may depend on home environment, emotional stress, and physical activity of everyday life, which is why home-based titration could be beneficial. Little is known about the indication for home-based titration and how this can be managed in an optimal and safe way.

Parkinson's disease (PD) is a progressive neurodegenerative disorder affecting a wide range of motor and nonmotor functions, leading to marked disability in its later stages [2]. Common motor symptoms in PD such as tremor, bradykinesia, and rigidity cause, in combination with the burden of NMS, a major impact on quality of life (QoL), ability to stay independent, and sense of control of the symptoms [3, 4].

The goal of medical management of PD is to provide symptom control for as long as possible while minimizing side effects [1, 5]. Currently, the available treatment options for PD are symptomatic and do not slow the neurodegenerative process. Levodopa is currently the most effective symptomatic therapy for PD, but, despite high-frequency oral dosing, fluctuations in motor symptoms and NMS still occur, affecting QoL, activities of daily living (ADLs), and social activities [6–8]. With time, most PwPs will need advanced therapy aimed at stabilizing symptom fluctuations and minimizing off-periods [8].

The decision to transition to advanced therapy is complex. When choosing a treatment, the best available evidence should be combined with the professional's expertise and the patient's needs and preferences according to the best practice of shared decision-making [9]. Finding an optimal dose for each patient requires dose adjustments based on the patient's response to treatment and score in the motor function scales. This can take multiple steps to achieve why admission over a week is traditionally needed according to clinical experience [9, 10]. Levodopa-carbidopa intestinal gel (LCIG) represents an effective option for continuous infusion in advanced PD [10, 11]. The treatment offers continuous intestinal levodopa infusion into the duodenum via a portable pump and a surgically implanted tube (jejunal extension tube, PEG-J tube) [12]. Prior to this procedure, a nasojejunal LCIG testing is conducted. For optimal effect, the LCIG dose is individually titrated, and in a Danish context, the testing is traditionally conducted in an in-hospital setting for a week in a standard neurological department, including nasojejunal LCIG testing, which is not standard procedure in many other comparable countries.

However, clinical experience shows that PwPs are often very stressed during the hospital stay due to noise, impaired sleep, and unfamiliar surroundings [12, 13]. These conditions make fast and effective dosage titration difficult, and the dosage often needs to be recalibrated once the patient is back home in familiar surroundings [11, 12]. Furthermore, hospitalization is very costly, and home-based titration may reduce healthcare costs by reducing the need for hospitalization and bed occupancy, as well as HCP and patient time. Organization of treatment, reimbursement systems, titration programs, and technological possibilities are country-specific, which is why these contextual factors must be considered in changing a standard treatment regime [12]. The use of telemedicine (TM) and video communication systems may offer an alternative approach, e.g., allowing LCIG initiation and titration procedures at home. In a previous study from 2017, it was reported that TM-assisted LCIG titration at home was resource-efficient, technically feasible, and well-accepted and was deemed satisfactory by patients, neurologists, and nurses [14]. These factors were the basis for the present home-based titration study as this procedure may allow optimized tailored therapeutic regimens and satisfactory treatment outcomes for PwPs and their caregivers. However, the Swedish study also emphasizes the importance of finding appropriate and motivated PD patients to use TM for home titration [14], which is why the establishment of clinical and patient profile characteristics is a secondary objective of the present study.

The primary objectives were as follows: firstly, to assess the feasibility and safety of LCIG home-based titration and patients'/caregivers' satisfaction, and secondly, to establish practical recommendations for home-based titration, including characteristics for PwPs suitable for home titration, and identify barriers and facilitators. It is hypothesized that home-based titration is feasible and safe, can lead to greater quality in the treatment, and is rated satisfactory for both PwPs and their caregivers.

## 2. Materials and Methods

### 2.1. Design

This study is an observational feasibility study assessing the feasibility and safety of home-based, TM-aided titration of levodopa duodenal infusions with the use of self-reported questionnaires.

### 2.2. Recruitment and Participants

All eligible participants were screened consecutively at the Movement Disorder Clinic (MDC) at Rigshospitalet, Denmark, from October 2017 to February 2022. Due to the COVID-19 pandemic, the inclusion of participants was delayed for almost 1.5 years. Eligible participants received written and verbal information from a neurologist specialized in movement disorders. Prior to PEG-J tube placement, PwPs were evaluated by the neurologist to determine whether they were appropriate candidates for LCIG, as indicated by the presence of 5 or more dosages of medication, 2 hours with “off” periods, and 1 hour with troublesome dyskinesia during the day despite optimized treatment with oral PD medications inspired by the “5-2-1” criteria [15, 16]. Those PwPs who declined to participate were offered normal titration during hospitalization. If included, the participants and their caregivers, along with home nurses and staff at nursing homes, were informed about the home-based titration program by a specialist PD nurse. Reasons for declining participation were registered systematically.

### 2.3. Sample

A total of 10 PwP candidates for advanced treatment were set as a clinically realistic goal for inclusion. Accounting for dropouts, a sample of 12–14 participants was estimated. To ensure diversity and representativeness, both PwPs living in private homes and nursing homes were recruited.

### 2.4. Inclusion and Exclusion Criteria

To be eligible to participate, individuals need to (1) be over the age of 18 years, (2) be diagnosed with idiopathic PD [1], (3) have the Hoehn and Yahr (H&Y) stage of PD = 3–5 [17], (4) be candidates for duodenal infusion treatment, (5) be independent or have access to support in their homes, (6) feel safe about the concept of home-based treatment and telemedicine, and (7) speak and understand Danish.

PwPs could not be included if they were (1) not eligible for advanced treatment, (2) diagnosed with severe depression, (3) suffering from untreated psychosis/hallucinations, and (4) had signs of dementia evaluated by the Montreal Cognitive Assessment Scale (MoCA) [18] rating of <24 (unless he/she had a healthy and motivated spouse/relative as a cohabitant or was living at a nursing home).

### 2.5. OpenTele System

OpenTele is a platform of telemedicine consisting of a server and a client on an Android-based tablet [19]. It consists of two large screens, a set of headphones with a microphone, checklists, and medicine devices for demonstration. During the video consultations, the neurologist or PD nurse asked about well-being, fluctuations, scores on standardized monitoring forms, use of medicine, and technical problems. The meeting typically lasted between 15 and 25 minutes. Technical problems associated with TM contacts related to the digital link were registered. The system was sponsored by external funding.

### 2.6. Procedure of Home Titration with TM

**Figure 1:**
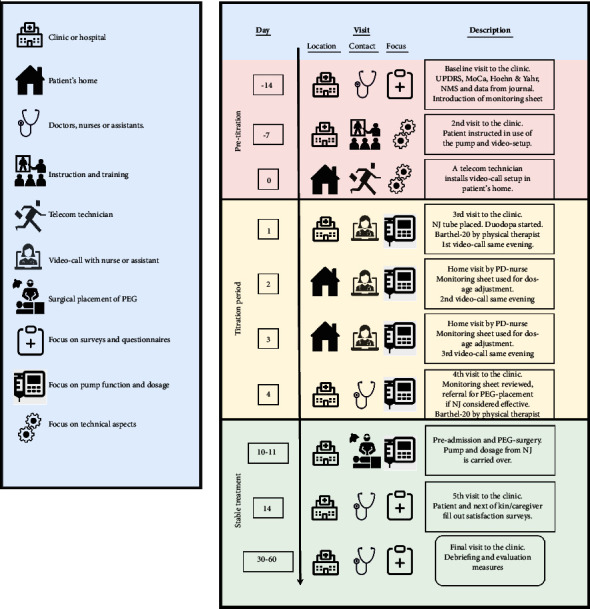
Overview of the contacts and days during the home titration period. Each visit, contact, and focus are visually presented in the legend to the left. (UPDRS = Unified Parkinson's Disease Rating Scale and NJ = nasojejunal tube).

A schematic overview of the home-based titration program (HBT program) is presented in Figure 1. The home titration and test of LCIG were estimated to last 4 days in total. A PD nurse visited PwPs in their own homes one time per day. Subsequently, a minimum of one video call was conducted depending on the participant's different needs.

#### 2.6.1. Pretitration

Contacts 1 and 2 (MDC, days −14 to −7): PwPs signed informed consent forms. All baseline measures were obtained (Figure 1). PwPs and caregivers were instructed to fill out the monitoring forms. Furthermore, information about both the LCIG pump and the use of the TM solution, OpenTele, was given. If the participants were living at a nursing home, the PD nurses from the MDC visited the location and instructed the staff.

Contact 3 (PwPs' Private Homes, day 0): TM system (OpenTele) was installed by a technician before the LCIG titration period at each patient's home. Instructions on the use of OpenTele were provided during the installation process. Also, if required, further technical support was provided by technicians during the home titration period.

#### 2.6.2. Titration Period

Contact 4 (MDC, day 1): The nasojejunal tube was placed for testing LCIG treatment prior to permanent PEG-J tube placement and identifying the relevant dosage before implantation and connection of the pump. The neurologist calculated the initial LCIG dose using a standardized formula [20]. Individually optimized dosing of LCIG was delivered over a 16-hour period, administered as a morning bolus followed by continuous infusion, and if needed, intermittent extra doses were determined by the PD nurse.

LCIG treatment was started in the MDC. During the stay, PwPs' functional status (Barthel-20 Index) was evaluated by a physiotherapist. During the observation period, follow-up home visits were scheduled and self-monitoring forms were handed out, enabling PwPs and relatives/staff at nursing homes to monitor symptoms during the titration (once an hour). If no side effects (e.g., hallucinations and dyskinesia) occurred, PwPs returned home.

Contacts 5–9 (PwPs' Private Homes, days 2–4): Initial dosage titration of LCIG was done in PwPs' own homes, supported by one visit from nursing staff and one video consultation a day. The objective of the first visit was not to achieve the optimal dose but rather to achieve therapeutic benefit with further fine-tuning performed by the patient and the clinician in collaboration. The PD nurse also assessed the effect of LCIG treatment based on the self-monitoring form and made dosage adjustments if necessary. Video calls were conducted by the PD nurse or a neurologist later in the day, where PwPs were asked about the placement of the nasojejunal tube, potential difficulties, and management of the medication. If a scheduled TM session failed, it was replaced by a phone call. Extra doses were adjusted individually during the titration period.

Both staff at the nursing homes and patients/caregivers had the possibility to ask questions via a support hotline to a nurse who specialized in tube care and pump management. Resource utilization including the overall number and duration of contacts per type of contact (e.g., video calls, home visits, and patient/technician involved in TM-related issues) was registered.

Contact 10 (MDC, days 10-11): On arrival at the MDC after home titration, a brief motor examination was completed to provide a point of comparison for assessing response to therapy (Barthel-20 Index, evaluated by a physiotherapist). The self-monitoring forms were evaluated by the PD team and the PwP. Based on these, it was decided whether the PwP was a candidate for a permanent jejunal extension tube.

#### 2.6.3. Stable Treatment Period

Contact 11 (Surgical Department): The jejunal extension tube is inserted (standard practice) at the surgical department. When the PEG/J tube position was confirmed radiologically, the patient returned home.

Contact 12 (MDC, day 14): Postsurgical follow-up includes stoma and complications. Both PwPs and caregivers received patient-reported evaluation measures (PREM) questionnaires to complete (Supplementary Files S1 and S2).

After the program, the patients followed the routine consultations, including registration of motor fluctuations and adjustments in medical treatment.

### 2.7. Data Collection

The following descriptive data were collected at baseline: age, sex, years with PD, H&Y stage [17], cognitive status (MoCA) [18], Barthel-20 Index (version 30.11.2017), comorbidity (Charlson's Comorbidity Index), cohabitant status, and levodopa equivalent dose (LED) calculated using Tomlinson's table [20].

### 2.8. Primary Outcome

#### 2.8.1. Patient-Reported Evaluation Measures (PREM)

The primary outcome was scored on PREM questionnaires (Supplementary Files S1 and S2) developed specifically for this study. Satisfaction with treatment was assessed for both patients and caregivers 14 days after the end of titration. PwPs and caregivers were asked ten questions separately on a scale ranging from “highly satisfied” to “very unsatisfied.” The scores were calculated by summing up the number of 10 answers: “highly satisfied” = 4 points, “satisfied” = 3 points, “unsatisfied” = 2 points, “very unsatisfied” = 1 point, and “don't know” = 0 points (not included in the total score). The scale then ranged from 0 to 40 points (Figures S1 and S2).

#### 2.8.2. Measurements during Titration

Hours with dyskinesia per day and hours with off-time per day were self-reported by the PwP or the caregiver on the evaluation forms.

The average time used by PD nurses and neurologists was calculated from baseline (start of LCIG treatment) until the end of titration, defined as the number of minutes the healthcare professionals (HCPs) had contact with the participant visiting their home, assisting with TM equipment, or by telephone.

The average number of contacts with HCPs was counted and summarized from baseline until the end of titration. Contacts in total were defined by TM support, telephone, home visits, and others during the titration period.

#### 2.8.3. Clinical Measurements

Clinical measures were obtained at baseline and are included in the characteristics of the patient profile (Table 1). The *Unified Parkinson*'*s Disease Rating Scale Part III (UPDRS-III)* [21], with scores ranging from 0 (asymptomatic) to 132 (most severe disabilities), and the *Non-Motor Symptom Questionnaire (NMSQuest)* [22], a 30-item self-completed questionnaire, with scores ranging from 0 to 95, provided clinical data on the severity of the burden of both motor symptoms and NMS in each individual.

#### 2.8.4. Analysis

Continuous data were summarized using descriptive statistics (means, standard deviation, minimum, and maximum), and categorical data were presented using frequency and percentage. Descriptive data were based on the number of included persons from baseline, including baseline data/observations from the two dropouts.

#### 2.8.5. Ethics

The study was granted ethical approval from the Capital Region Research Ethics Committee, Copenhagen, and was registered at ClinicalTrials.gov (study identifier NCT4196647). Written informed consent was obtained from all participants.

## 3. Results

**Figure 2:**
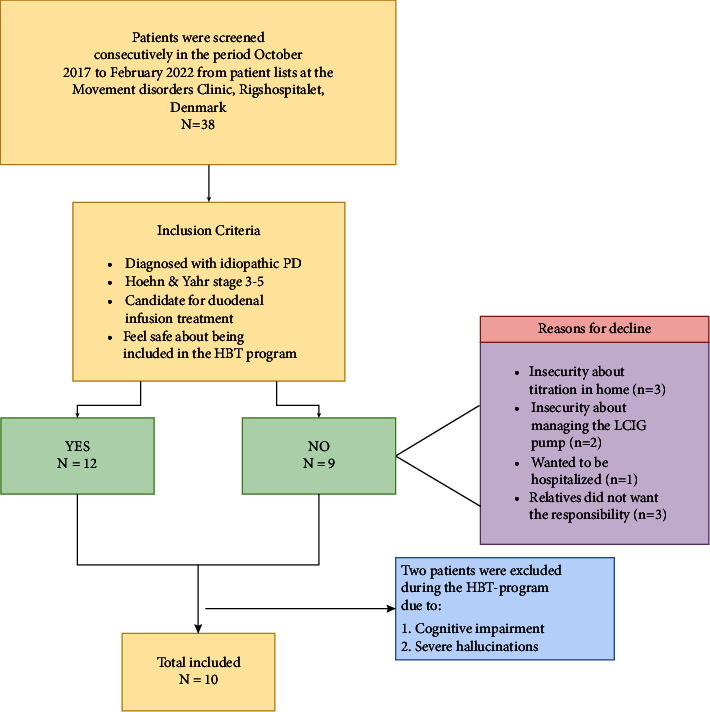
Flowchart of the recruitment process and dropouts.

A total of 12 PwPs of 21 eligible candidates were included in the study, and 10 fulfilled the HBT program. Four major reasons for declining participation were identified and are listed in the flowchart (Figure 2).

In Table 1, all data are presented descriptively to characterize the cohort.

The primary outcome, assessed on the PREM questionnaire, showed that PwPs (mean 36.2) were on average more satisfied with the HBT program than caregivers (mean 31.8).

Baseline demographics revealed a varied spread of descriptive and clinical indicators of the 10 included participants. Of the 12 baseline completers with a mean age of 74 years (range 64–81), 5 were female (42%) and 7 (58%) were male. The average time in years with PD was 12.9 years (range 6–20), and the average H&Y stage was 3.2. The study cohort had a low degree of comorbidities and a relatively good function level (Barthel scale, mean 17.2). However, 8 of 12 were dependent on help, classified by either living at a nursing home or receiving professional help in their own homes. These 8 PwPs also had a high burden of NMS. MoCA scores varied between 16 and 30 points. As expected, all participants required high levels of LED before inclusion in the study. The measurements during titration and PREM data were only obtained on 10 PwPs completing the study. The time spent with home visits for HCPs was on average 93.4 minutes (ranging from 35 to 180 minutes), and the length of the titration period lasted on average 3.4 days. All participants were offered 3 video calls during titration, but some were changed to a phone call instead due to technical challenges with the TM system.

Facilitators and barriers were identified based on the descriptive results and the reported notes from PwPs and caregivers in the open-ended questions of the PREM questionnaires (Table 2).

## 4. Discussion

In this feasibility study, we synthesize both the types of indicators associated with the conduction of the HBT program, characteristics of eligible PwPs, and facilitators and barriers that may influence its implementation. The main facilitators for the implementation of an HBT program were primarily related to internal organizational factors in the MDC, multidisciplinary collaboration, and skill mix of professionals to support both PwPs and caregivers, whereas barriers were linked to challenges with the TM system, eligibility criteria (complexity and social situation of the patient), and educational aspects. From a clinician perspective, neurologists and nurses commented that the program made it “more easy to balance the right doses,” but they also reported that they “didn't have the same sense of control” of the titration process.

Results suggest that the program is feasible and safe, and PwPs can be included in the program even though they are cognitively impaired as long as they have a healthy caregiver/cohabitant, who possesses the mental capacity to adjust to technical and unforeseen challenges. Our results show that both the participating PwPs and caregivers felt safe and were satisfied. However, the average score in the evaluation PREM questionnaire was lower in the group with relatives (mean 31.8) than the PwP outcome (mean 36.2), which may indicate that a lot of pressure and responsibility are put on caregivers in this process. Moreover, both experience from the inclusion process and results from PREM questionnaires indicate that caregivers are the primary decision-makers when it comes to implementing an HBT program. Therefore, when feasible for LCIG treatment, PwPs and caregivers should be involved in these discussions about home-based titration, particularly if the caregiver needs to assist in the setup and management of the program and TM system and provide reliable information regarding symptoms. Few clinical studies have been made with PwPs and home-based treatments for other chronic diseases, but an extended focus on caregivers cannot be found in other similar studies.

A meta-analysis from 2021 concludes that chronically ill patients who underwent home-based interventions may be as safe as hospitalization with no difference in mortality and, in fact, a lower risk of readmission and long-term care admission [23]. However, the meta-analysis also showed that the length of treatment was longer in patients with chronic illnesses undergoing home-based treatments than in-hospital admissions. Our results differ from these conclusions as the LCIG treatment was sufficient and rated safe by PwPs and caregivers within a period of 3.4 days on average compared with the standard in-hospital week. However, time spent with home visits differed from individual to individual (range 35–180 minutes), indicating very different needs of physical contact of support. In addition to that, the positive effects of the HBT program may not solely be related to the medical therapeutic effect but also to the empowerment initiation, increased responsibility, and better relationship with treating HCPs as experienced by the PD team at the MDC. It was mainly technical issues that determined the time consumed and difficulties managing the LCIG pump. A recent study reported similar findings, where the median time spent titrating LCIG was 3 hours (range 0.5–5 h) and mostly due to TM-related issues [24].

The complexity and multifaceted nature of PD requires an extended understanding of workflows across sectors, and with an aging population and the growing prevalence of chronic diseases affecting all age groups, the integration of home care services and alternative treatment interventions is becoming a necessity for front-line health service organizations [25–27]. As hospitalization is one of the key factors in the increasing cost associated with the use of health services related to chronic diseases, it is essential to implement effective and safe alternatives to conventional hospitalization [28]. Moreover, early studies in the implementation of telemedicine and its effect on HCP's work practice show that telemedicine initiatives should be approached with a special emphasis on educational and organizational considerations [29]. Hence, an elaborated “cross-sectoral understanding” of different workflows and contextual factors related to the different sectors is vital.

Even though PwPs and relatives rated the process with great satisfaction and seemed to be empowered by the HBT program, some of the self-reported monitoring forms were not adequately filled out and revealed subjective perceptions of response to treatment and side effects, which could be unreliable [30]. Therefore, it will be advantageous in a future setting to involve measurements of motor symptoms with wearables, such as sensors, accelerometers, and other algorithm data obtained during the titration period [31, 32]. In that case, even more emphasis should be put on educational aspects in implementing an HBT program for both cross-sectoral HCPs and PwPs/caregivers.

Hospitalizations are costly and may lead to adverse events [28], and hospital-at-home interventions could be a substitute for some in-hospital stays. However, there may be several differences between countries in terms of both local resources and legal issues regarding home-based treatments. A recent scoping review reveals that differences may depend on identified person-level and system-level barriers. The person-level barriers included the cost of services. The system-level barriers included the availability of appropriate healthcare resources [33]. Despite the increasing number of home-based interventions concerning PD care, health economic evaluations are rare and the results vary greatly [34]. Therefore, there is a need for flexibility and adaptation of this approach to accommodate the unique circumstances and regulatory constraints of different countries. Strategies such as remote telecommunication support and alternative models of care delivery may be explored to overcome these challenges and facilitate the implementation of home-based LCIG titration in diverse healthcare settings.

Taken together, our findings provide a mapping of indicators of the HBT program categorized by the factors identified as barriers or facilitators to its implementation.

### 4.1. Limitations and Future Directions

This study has some limitations, particularly regarding the potential generalization of results. Due to the small sample size, we can only generate hypotheses and evaluate feasibility and applicability. However, the results have the potential to classify facilitators and barriers and descriptively indicate eligible candidates for future implementation of the HBT program.

The PREM questionnaires were developed specifically to evaluate the degree of satisfaction of PwPs and caregivers in this study, making it possible to tailor the questions and not evaluate the HBT program on a generic instrument. However, some of the questions could have been misunderstood as they were not face-validated prior to the study.

In a future study, commencing in June 2024, we plan to conduct a randomized controlled trial (RCT) to assess the impact of the HBT program compared with standard treatment and hospitalization. The insights gained from the overall results of this feasibility study will inform the design and implementation of the upcoming RCT. Additionally, a cost-effectiveness analysis to provide a more robust assessment of the economic implications of our approach will be included.

## 5. Conclusion

In conclusion, the findings from this real-life feasibility study indicate that telemedicine (TM)-assisted home-based titration (HBT) programs are feasible and also rated as satisfactory and safe by PwPs and their caregivers. These programs have the potential to serve as an alternative to in-hospital treatment. Feasibility was demonstrated by the number of contacts, the amount of time spent in PwPs' private homes (tailored to individual needs), and the quality of TM-assisted interactions. The safety and satisfaction of both PwPs and caregivers were evident. However, it is crucial to provide extended focus and support to caregivers in both decision-making processes and throughout the HBT program. Furthermore, this study identifies indicators of a PwP profile suitable for an HBT program and highlights facilitators and barriers to implementation. These insights underscore the need for clinically adaptable approaches in tailoring HBT programs to the unique needs and circumstances of PwPs and differences in regulations and resources between countries. By addressing these factors, future HBT programs can be optimized to maximize patient satisfaction while ensuring caregiver support and safety.

## Figures and Tables

**Table 1:** Descriptive data (including the 2 dropouts) and clinical characteristics of all the participants using descriptive statistics.

Descriptive data	Mean/SD	Min/max
Age	74.0/5.0	64/81
Years with PD	12.9/4.6	6/20
MoCA score	23.1/4.3	16/30
Barthel score (before titration)	17.2/2.8	11/20
Charlson's Comorbidity Index score	3.25/1.2	1/6
Levodopa equivalent dose (LED) at baseline	1187.2/356.3	550/1850
LED at the end of the HBT program (day 14)	1097.6/302.5	565/1578

Hoehn and Yahr scale score	*N*	%

H&Y = 3	8	75
H&Y = 4	4	25
Sex		
Female	5	42
Male	7	58
Housing status		
Living alone	2	17
Cohabitant	8	66
Nursing home	2	17
Daily function		
Independent of help	4	33
Dependent on help	8	67

Clinical variables at baseline	Mean/SD	Min-maximum

NMSQuest score (0–95)	67.5/22.3	43/81
UPDRS-III score (0–132)	43.6/16.4	27/73

Evaluation outcomes	Mean/SD	Min-maximum

Primary outcome		
Evaluation score (PwPs) (0–40)	36.2/3.9	27/40
Evaluation score (caregivers) (0–40)	31.8/7.2	25/39
Secondary outcomes		
Hours with dyskinesia (per day) during titration	2.4/1.8	0/5.3
Hours with off (per day) during titration	8.7/4.1	4/15.5
Time (minutes) spent in PwP's home (HCP)	93.4/35.2	35/180
Number of video or phone calls to PwP	3.2/0.3	3/4

**Table 2:** Classification of facilitators, barriers, and a PwP profile in implementing an HBT program.

*Facilitators*
(i) An HBT program is feasible due to the length of the titration period, the number of contacts to MDC, and the time spent in private homes
(ii) Timeframe of 3–4 days of home titration is sufficient
(iii) The HBT program is experienced as safe for PwPs and caregivers as long as they have a “hotline” to MDC/support nurses and TM assistance when needed
(iv) Caregivers are primary decision-makers and hold the responsibility during the titration process, which is why they must be actively involved and informed during the process
(v) An environment for a daily and close dialog between the HCP members in the PD team is recommended

*Barriers*
(i) Technical improvements in the TM system and earlier introduction should be considered to improve digital competencies of PwPs and caregivers
(ii) The program has to be established with educational initiatives for all parties: PwPs, caregivers, and cross-sectoral HCPs in primary care before starting
(iii) A vast amount of responsibility is placed on the caregivers, which must be mitigated by support from the PD team and the support nurse

*PwP profile eligible for HBT*
(i) Cognition is not a vital parameter if the PwP has a caregiver, who is healthy and motivated to participate
(ii) Living at home or in nursing home (more education to HCPs in primary care is needed)
(iii) Capable of managing a TM system
(iv) Are motivated and feel safe with home treatment
(v) No severe side effects of LCIG (hallucinations and psychosis)

## Data Availability

The authors confirm that the data supporting the findings of this study are available within the article. Derived data supporting the findings of this study are available from the corresponding author (THT) upon request. However, the authors are restricted by the data sharing policies in the Capital Region, Denmark, so data sharing has to be authorized by the data agency.

## Supplementary Materials

Supplementary Materials Supplementary Files S1 and S2 include the patient-reported evaluation measures (PREM) questionnaires for both relatives and included PwPs.
