# Supplementary material for: Home-Based Titration with Duodenal Infusion of Levodopa-Carbidopa Intestinal Gel in People with Parkinson's Disease: An Observational Feasibility Study
Source: Parkinsons Dis. 2024 Apr 8;2024:5522824. doi: 10.1155/2024/5522824 (PMC11018374; doi:10.1155/2024/5522824)
Supplement: Supplementary Materials — Supplementary Files S1 and S2 include the patient-reported evaluation measures (PREM) questionnaires for both relatives and included PwPs. [file 5522824.f1.zip › Home titration PREM S1.docx]

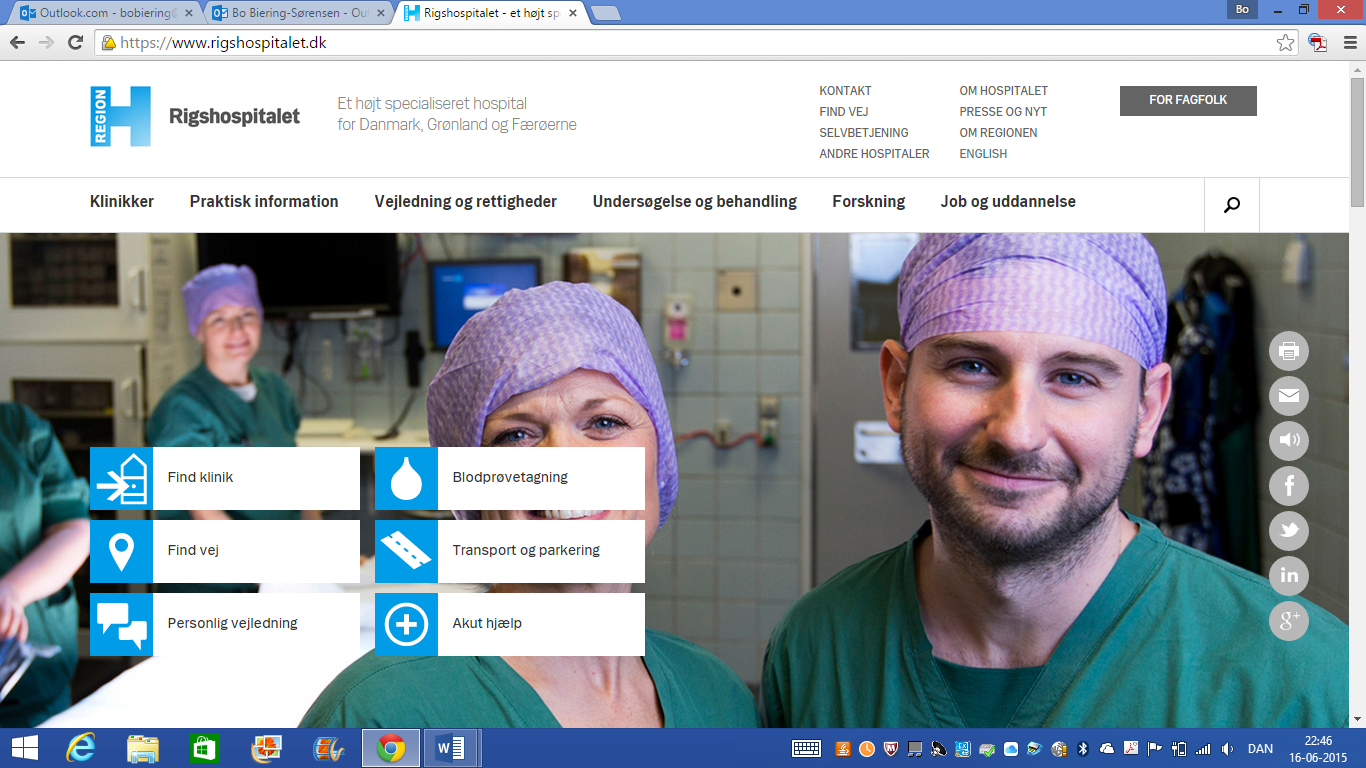


**Hometitration Evaluation**

**Patient cpr: ___________________________________________**

**Start date & year: _____________________________________**

**End date & year: _______________________________________**

**D**

***Next of kin Satisfaction Survey:***

1.Before agreeing to home-titration; did you feel well informed about the process of home-titration with Duopopa?

Yes, to a high degree □ Yes, to some degree □ Yes, to a lesser degree □ No, not at all □ Don’t know □

2. Have you felt that testing Duodopa for your partner in your own home was safe?

Yes, to a high degree □ Yes, to some degree □ Yes, to a lesser degree □ No, not at all □ Don’t know □

3. Did you feel that the Parkinson-nurse was sufficiently present in your home, during the trial period?

Yes, to a high degree □ Yes, to some degree □ Yes, to a lesser degree □ No, not at all □ Don’t know □

4. Did you receive sufficient instruction and training about introducing and stopping the Duodopa pump treatment?

Yes, to a high degree □ Yes, to some degree □ Yes, to a lesser degree □ No, not at all □ Don’t know □

5. Was it easy to use the videocall/tablet to register your partners movements?

Yes, to a high degree □ Yes, to some degree □ Yes, to a lesser degree □ No, not at all □ Don’t know □

6. Was it easy to use the video/tablet for telemedicine/communication with the Parkinson-nurse?

Yes, to a high degree □ Yes, to some degree □ Yes, to a lesser degree □ No, not at all □ Don’t know □

7. Have you been satisfied with being able to have the testing and titration of Duodopa be conducted in your home?

Yes, to a high degree □ Yes, to some degree □ Yes, to a lesser degree □ No, not at all □ Don’t know □

Kan smerteskolen med de informationer der gives til patienter og pårørende gøre dagligdagen nemmere? Ja □ Nej □

Kan information og træning via smerteskolen give CRPS patienter højere livskvalitet? Ja □ Nej □

Har du forslag til hvordan smerteskolen kan gøres bedre eller kommentarer til ovenstående spørgsmål (skriv evt. videre på bagsiden:

____________________________________________________________________________________________________

**THANK YOU for participating in home-titration.**

Movement Disorder Clinic, Rigshospitalet, Glostrup

8. Have you been satisfied with your partner not being in hospital for the testing and titration of Duodopa?

Yes, to a high degree □ Yes, to some degree □ Yes, to a lesser degree □ No, not at all □ Don’t know □

9. Has your partner been satisfied with titration being conducted in your own home?

Yes, to a high degree □ Yes, to some degree □ Yes, to a lesser degree □ No, not at all □ Don’t know □

10. Would you recommend home-titration to other patients with Parkinson’s Disease and their next of kin who are trying Duodopa?

Yes, to a high degree □ Yes, to some degree □ Yes, to a lesser degree □ No, not at all □ Don’t know □

Do you have suggestions for how home-titration can be improved, or comments regarding the questions above? (continue on back of page if needed)

____________________________________________________________________________________________________

____________________________________________________________________________________________________

____________________________________________________________________________________________________

____________________________________________________________________________________________________

____________________________________________________________________________________________________
